# Supplementary material for: Patterns of Intron Gain and Loss in Fungi
Source: PLoS Biol. 2004 Nov 30;2(12):e422. doi: 10.1371/journal.pbio.0020422 (PMC532390; doi:10.1371/journal.pbio.0020422)
Supplement: Table S1 — Also available at http://genes.mit.edu/NielsenEtAl/. (4.3 MB ZIP). [file pbio.0020422.st001.zip › NielsenEtAl/html/1436.html]

AN9059.1.NCU02379.1.MG03552.1.FG06363.1


```
 CLUSTAL W (1.82) Multiple Sequence Alignments - Introns Inserted


Sequence 1: NCU02379.1	637 aa
Sequence 2: MG03552.1	582 aa
Sequence 3: FG06363.1	641 aa
Sequence 4: AN9059.1	477 aa
Alignment Length: 665 aa
Number Identitical Residues: 93 aa
Alignment Score (without introns) 7488


MG03552.1 	-----MLGTIPDEIVSHILQYVPPEDNLRCFQYLSRRLQRLANEELLWRSHCCLCFKYWD
NCU02379.1	MTSSSSLDRFPDELIRHILLYVSPEDNVLNIQPVSRRFYHIANEPLLWRYLCRNSFTFWE
FG06363.1 	----MSLLQIPDEIIQHLLYYIPPEDNLSSFQFLSHRLRHLANESLLWRYHCQSSFTFWN
AN9059.1  	------------------------------------------------------------
          	                                                            

MG03552.1 	AQHDFKGKLK-LKVKEVDWKKLWILRRRQNERVLHLLDGILESKVGRLQKFEKICLLGYD
NCU02379.1	PEHCFFAKLSDPRPTTIEWKKLWLRRKRQNHLIKRLLDEILTTRYHQLKNLQRICQFGYD
FG06363.1 	PEHNFYRRLK-GRASSTPWKKIFLIRKSRNAQVERLLVEILETKVGRLKRFEKVCKFGYD
AN9059.1  	-----------------------------------------MSQTGRIEKFRSIVSFGYD
          	                                          ::  :::.:. :  :***

MG03552.1 	AKDLLLEQCHVDESVEDVLARR~YHANAALGSIHRSMAVKEWSKFQQPYSGPRP------
NCU02379.1	AKDFLLEQCHVDESHDDVLARR2YYANSALDSIHRGMAVEVWSKCYETGTDPYSG-----
FG06363.1 	AKDFLLEQCNADETAEDVLARR2YYSNSLLDSIHRSLAIEQWYNIQQASRDTSRHPANLS
AN9059.1  	AKDTLLRNISIDSSAEDYLARR2YYARALLTSLHRTIAIPAWASLRDGNEISLAN-----
          	*** **.:   *.: :* **** *::.: * *:** :*:  * .  :    .        

MG03552.1 	LERALAGFDMFVLHDQEGDIDD0IQN------------------ALDRYSRMFLESCPNF
NCU02379.1	LDRALGAFDLFVLHDQPQDLEY0ILS------------------TLDTLAQRFMLEHPEH
FG06363.1 	LERALGAFDLFVLHDQPGDLDD~VSASYPCSGHTPVNDSIKIGLILDRLAADFRDTQPNI
AN9059.1  	---ALGAFDLFIPESGFGNFDE0ISAR------------------LNQILSGCLLEFPDI
          	   **..**:*: ..   :::  :                     *:          *: 

MG03552.1 	KELTTREKARALLSWVRSENLTGMEDPATNYRNIRNCLIGHALLDEGHPSLPLISSAIYS
NCU02379.1	KIMSTRQKALALNRWLRAQNLMGMENETVNYHNLRNCLLGHALSDDEHPSLPIISSAIFA
FG06363.1 	DGMSTRQKALELNRWLRWNNLTGLQHPERSYRNLRNCLIGQALRHEDHDSIPIISSAIFC
AN9059.1  	QNLSPRRKASAIAAYLRSHNMTGIG-PGREYHCLEHNFLGIALNDPAHNSLPLISAAIYC
          	. ::.*.**  :  ::* .*: *:     .*: :.: ::* ** .  * *:*:**:**:.

MG03552.1 	ALAQRVGLRSECCAFPAHVHASVFAPSNETLDGTPVADPTAELEVMYLDPFESDHEINVE
NCU02379.1	SVAERLGMSSACCAFPSHVHASVKAPPGMTLDGDVEEDPKAEPETMYLNPYKWDGEVTLD
FG06363.1 	CVAERLGLQAQCCAFPTHVHAMVFAENGKTLDSVPVTEDDAPLERMYLDPYGSSEEIPMA
AN9059.1  	HVAQMLGLDARPCGFPFHVHVIVIPPSGFDIDGNPITTQ---GEPIYMDPFRSDQETPAS
          	 :*: :*: :  *.** ***. * .  .  :*.          * :*::*:  . *    

MG03552.1 	DLRMRLEDFGWE-NGRDAFLRASPVPVLVNRTAHNIRVSYVRYQHSSD-----VEAAKFS
NCU02379.1	ELRRALVELGWT-QGSEVFLRASPVPIIVLRTAANIKAATSRIQNLADRSGEPIEVEAKR
FG06363.1 	DLRSMLAHFGWQ-TSTDKFLSPVSPVTIAMRTARNIRATASRVIEAREQ----ADPDLTR
AN9059.1  	DLRTQLNFLGASPIEQVAFMGESETSEIVLRCSKNILNSIQRRNQSSS------------
          	:**  *  :*  .     *:       :. * : **  :  *  .  .            

MG03552.1 	FRSGDPFHNMEALLYSVLWVSLLVT-PTTYQLWDNNLEHFLNRFHKYFSEDAWMVRKYLA
NCU02379.1	LRAGHPDINVEAAKYASMWAELMVK-PVSSVHWDHNLETFLHSFAMSWGEDAWIVRKYLL
FG06363.1 	LITGNDSSNIDAALYSALWASLLLT-PVDSFEWDEALEPFLNRFAKSWHVDAWLVEKYIF
AN9059.1  	-------IDAVCARYAALWSSLLLSNHLRPAELQHHLLWLMELVATDFPCDIHLVERYIA
          	        :  .  *: :* .*::..       :. *  ::. .   :  *  :*.:*: 

MG03552.1 	PLYDEFAATHGRYGAAARSSWEDVHQTLDMEHNLDMRIPTVSRRYTQAIQKSVRYRVGQV
NCU02379.1	PMYEQFVASQP--HVRNRAGWENVREILNMLDNLDRREAVVTRRYTQEMQERVRYRIGQV
FG06363.1 	PLYDRFGPLRERIMRNNPRRWDDPREVINLVDEFDEVPPPVFRRNSVRTQH-VLYKIGQV
AN9059.1  	SLFR------------GTPEVEHILESLHVIRAVDMIPKQVKRR--SFEHKSVKYHIGQV
          	.::                  :.  : :.:   .*     * **     :. * *::***

MG03552.1 	FVHKRYGFCSIINGWTADGVSSIPNPHVLP--FDEGHNPAAQEDNA~GG-----------
NCU02379.1	FRHKRYGWIGIINGWAAG-SAGLPVPHYLDGYEDEVQSPTTSPRSS~GDRLHSEGQLRPQ
FG06363.1 	FKHRRYGWIGAVNGWTDQGTRRLPMPHTVAIDETLDDNSDTELPNR~LRPRN--------
AN9059.1  	FRHRRYNYTAIITGWDAECGAGEQWMRRMGIDHLQSGRHQSFYHVI2VEDKS--------
          	* *:**.: . :.**           : :           :                   

MG03552.1 	---------~-----------VTPNSQE--TDGSASGE----ITGPSGGTRDPISQIFYT
NCU02379.1	LARVFYTCM2SSKRPRIDRLRVAQNSIEIITDPNLIPESLKFLAGKYFKRFDRKTCTFVS
FG06363.1 	--KTFYTCL2RTTGP--ERHVVAEDNIVLIEDPSEIPDSLFRQAGKFFKRFDAETCTFVS
AN9059.1  	---------~-------VRYVAEENIQLISPTVSELSPTLVAVAGKYFKRWDSSSREFVS
          	                     .  :        .    :    :*      *  :  * :

MG03552.1 	CM-------
NCU02379.1	NIKESYPDD
FG06363.1 	NVTEQYPDN
AN9059.1  	NIKDEYPDD
          	 :... ...
```
